# Deciphering the Genetic Mechanisms of Salt Tolerance in *Sorghum bicolor* L.: Key Genes and SNP Associations from Comparative Transcriptomic Analyses

**DOI:** 10.3390/plants12142639

**Published:** 2023-07-13

**Authors:** Donghyun Jeon, Jin-Baek Kim, Beum-Chang Kang, Changsoo Kim

**Affiliations:** 1 Department of Science in Smart Agriculture System, Chungnam National University, Daejeon 34134, Republic of Korea; jemdong@cnu.ac.kr; 2 Advanced Radiation Technology Institute, Korea Atomic Energy Research Institute, Jeongeup 56212, Republic of Korea; jbkim74@kaeri.re.kr; 3 Department of Horticulture, College of Agricultural Life Science, Jeonbuk National University, Jeonju 54896, Republic of Korea; 4 Department of Crop Science, Chungnam National University, Daejeon 34134, Republic of Korea

**Keywords:** RNAseq, osmotic stress, non-synonymous SNP, Dhurrin, *Sorghum bicolor* L.

## Abstract

*Sorghum bicolor* L. is a vital cereal crop for global food security. Its adaptability to diverse climates make it economically, socially, and environmentally valuable. However, soil salinization caused by climate extremes poses a threat to sorghum. This study aimed to identify candidate salt-tolerant genes and single nucleotide polymorphisms (SNPs) by performing a comparative transcriptome analysis on a mutant sorghum line and its wild type. The mutant line was generated through gamma ray exposure and selection for salt tolerance. Phenotypic measurements were taken, followed by mRNA sequencing and variant calling. In this study, potential genes and non-synonymous SNPs associated with salt tolerance were inferred, including *LOC8071970*, *LOC8067721*, *LOC110430887*, *LOC8070256*, and *LOC8056880*. These genes demonstrated notable differences in nsSNPs in comparison to the wild type, suggesting their potential roles in salt tolerance. Additionally, *LOC8060874* (cyanohydrin beta-glucosyltransferase) was suggested as a key gene involved in salt tolerance due to its possible role in dhurrin biosynthesis under salt stress. In upcoming research, additional reverse genetics studies will be necessary in order to verify the function of those candidate genes in relation to salt stress. In conclusion, this study underscores the significance of investigating salt tolerance mechanisms and the potential key genes associated with salt tolerance in sorghum. Our findings may provide insights for future breeding strategies aimed at enhancing salinity tolerance and crop productivity.

## 1. Introduction

Salt stress is known to negatively impact crop growth and productivity. When soil salinity increases, it creates an osmotic imbalance that limits water uptake by plants and leads to ionic toxicity, resulting in reduced growth, decreased yield, and even death of the plant. As the salt concentration in the soil increases, the plant responds by activating various defense mechanisms at the molecular and physiological levels. For example, plants can synthesize compatible solutes, such as proline and glycine betaine, to maintain osmotic balance, and they can also activate antioxidant systems to scavenge reactive oxygen species (ROS) that accumulate under salt stress [1]. However, prolonged exposure to high salt concentrations can lead to irreversible damage to plant tissues, affecting various physiological processes such as photosynthesis, respiration, and nutrient uptake. For example, salt stress can result in a decrease in chlorophyll content, stomatal conductance, and CO_2_ assimilation, ultimately leading to reduced growth and yield [2,3,4]. At the molecular level, salt stress triggers the activation of various signaling pathways, including the mitogen-activated protein kinase cascade, the calcium-dependent protein kinase pathway, and the abscisic acid signaling pathway. These pathways mediate the expression of stress-responsive genes, such as those encoding transporters, enzymes, and transcription factors, which regulate various physiological and biochemical processes involved in salt stress tolerance [5,6,7].

*S. bicolor* is an important cereal crop that is widely cultivated around the world, with a global production of 57.6 million tons. It is valued for its drought tolerance and adaptability to a wide range of agro-climatic conditions [8]. Primarily used as food and feed, it has sustained millions of people in developing countries. Its considerable drought tolerance sets it as a vital contributor to global food security by offering a resilient alternative to other cereal crops susceptible to climate extremes. Sorghum’s environmental value lies in its ecological benefits, such as enhancing soil fertility, reducing soil erosion, and its potential as a bioenergy source. This crop’s cultivation and use trace back to thousands of years, playing a significant role in human civilization’s growth, especially in Africa and Asia [9]. Beyond its conventional uses, *S. bicolor* serves as an advantageous model organism for genetic and genomic studies of cereal crops. The ease of studying its diploid genome and the high natural genetic variation offers a rich pool of traits for crop improvement and breeding. Genome assembly and annotation, as well as transcriptomic studies under various conditions such as abiotic stress and pathogen infection, have yielded critical insights into its stress tolerance mechanisms and potential breeding targets [10,11,12]. These multifaceted perspectives on *S. bicolor*, spanning genetics, genomics, and transcriptomics, equip us with a comprehensive understanding of its genetic diversity and its potential for crop improvement. Information extracted from these studies can guide the development of new varieties with improved yield, quality, and resilience to environmental stresses, further enhancing its contribution to global food security [13,14].

Sorghum, a salt-tolerant species, employs adaptive mechanisms at the physiological, biochemical, and molecular levels to mitigate the adverse impacts of salinity stress. These include the maintenance of photosynthetic apparatus, osmotic balance, ionic equilibrium, and redox homeostasis [15,16,17,18]. Salinity-tolerant sorghum genotypes exhibit superior germination and growth parameters, maintaining efficient photosynthetic machinery and pigments under salt stress conditions [19,20,21]. These genotypes also possess the ability to regulate toxic and beneficial ion concentrations in response to salinity, thus ensuring ion homeostasis. This involves various membrane transport systems, which help retain essential ions and exclude or compartmentalize sodium ions [22]. Furthermore, alterations in cell wall constituents and cellular membrane structures play a pivotal role in the salinity adaptation of sorghum. However, the exact workings of membrane lipid remodeling in relation to sorghum salinity tolerance are not yet fully understood, necessitating further research [23,24]. Osmotic homeostasis, crucial for plant salinity tolerance, enables the regulation of internal water balance under saline conditions. This involves lowering the cellular water potential through accumulation of compatible solutes and/or inorganic ions, which helps maintain water absorption under saline conditions. Consequently, this osmotic adjustment is integral to sorghum’s adaption to salinity [25,26]. Finally, salinity stress disrupts cellular metabolism, leading to excessive reactive oxygen species (ROS) production. These ROS can damage proteins, nucleic acids, and other cellular components, causing membrane leakage. In response, plants activate antioxidant redox systems to control ROS homeostasis, a key adaptation mechanism to high salinity [27]. Understanding of sorghum’s salinity tolerance mechanisms is continually evolving, emphasizing the need for ongoing research in this area.

Mutation breeding has been effectively employed as an approach for enhancing crop diversity, particularly through the use of gamma-ray irradiation [28]. Numerous studies have highlighted the efficacy of gamma rays in improving the nutritional and functional characteristics of grain mutants [29,30]. For instance, sorghum mutants generated through gamma-ray exposure have shown increased concentrations of essential compounds such as amino acids [31], along with improved storage attributes due to decreased mold growth and free fatty acid content [32]. However, the technique has its downsides. While gamma rays are potent mutagens, their application could potentially lead to unexpected genetic changes, thereby raising stability concerns. Furthermore, mutation breeding often requires meticulous screening processes to identify favorable mutants, resulting in a labor-intensive and time-consuming process. Still, gamma-ray-induced mutation breeding has brought about significant advances, as shown in a study by [33], where 29 elite sorghum genetic resources were compared for their salt tolerance characteristics. More recently, a follow-up study developed 28 M6 mutant lines from eight original sorghum accessions/cultivars with enhanced salt tolerance [34]. Quantitative real-time polymerase chain reaction (qRT-PCR) analyses in these mutants revealed several genes likely involved in salt tolerance. Further, among 36 sorghum materials investigated, 10 gamma-irradiated mutants showed significantly increased biomass compared to their original accessions. Overall, radiation breeding, including gamma-ray-induced mutation, has been extensively utilized for generating novel genetic diversity, bypassing the long and arduous process of traditional breeding [35]. Such methods have been particularly focused on improving grain yields under environmental stress and enhancing biomass for the bioenergy industry.

This study performed a comparative transcriptome analysis of *S. bicolor* wild type and mutant lines with salinity tolerance to identify differentially expressed genes (DEGs) and nsSNPs that are crucial for salt tolerance, and also provides a better understanding of the genetic mechanisms underlying salinity tolerance in sorghum and identifies genes and SNPs that are associated with salt tolerance. Moreover, this study will be useful for improving sorghum breeding strategies aimed at increasing salinity tolerance and ultimately enhancing crop productivity.

## 2. Results

### 2.1. Germination Rate and Leaf Characteristics of the Wild Type and Mutant S. bicolor

We compared the growth information of the wild type and mutant *S. bicolor* prior to conducting the comparative genomic analysis. Before any stress treatment, we sowed 50 seeds each for the wild type and mutant S. bicolor, and recorded the germination rates. The germination rate of the wild type was 44%, while that of the mutant was 92%. This suggests that the mutant line had a more than two-fold higher inherent germination rate than the wild type, even in the absence of any salt stress. To investigate the number and length of leaves, we selected 15 intermediate-growing individuals for each type of sorghum and measured the mean values. The average number of leaves for the wild type was 4.8 ± 0.41 while that for the mutant was 4.93 ± 0.26. The average length of the longest leaf for the wild type was 30.25 ± 3.55 cm while that for the mutant was 32.15 ± 3.85 cm. We initially intended to document and compare phenotypic information post-salt treatment. However, we refrained from doing so because the stress treatment period was short (48 h) and was not sufficient to cause a significant growth difference. Therefore, no growth information was recorded after the salt stress treatment (Table 1). The leaf length and count showed slightly higher values in the mutant lines compared to the wild type, however, the differences were not statistically significant.

### 2.2. Generation of the Sequencing Data

**Figure 1:**
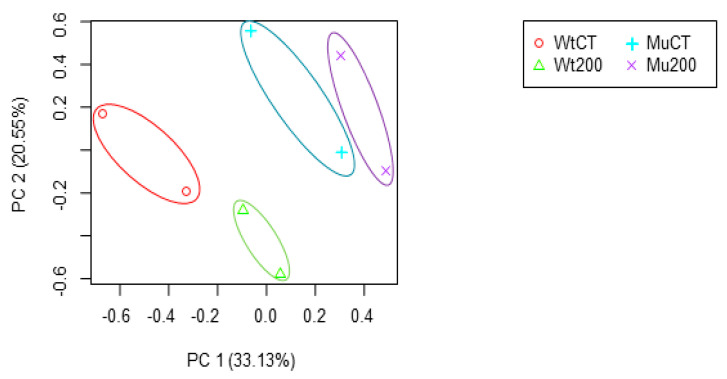
PCA of RNA-seq data for *S. bicolor*. The x-axis represents the first principal component (PC1) while the y-axis represents the second principal component (PC2). WtCT, Wild type control; Wt200, Wild type 200 mM NaCl treatment; MuCT, Mutation Control; Mu200, Mutation 200 mM NaCl treatment.

The RNA was extracted from the leaf tissues of *S. bicolor* (IT124115) and a gamma ray mutant line (IT124115M) to conduct mRNA sequencing. To examine transcriptional expression patterns under salt stress treatment, plant materials in the experimental group were treated with 200 mM NaCl, while the control group was not exposed to salt stress. Through two biological replicates, a total of eight sequencing datasets were produced. Before initiating a thorough analysis of the RNA-seq data, we conducted a Principal component analysis (PCA) to understand the correlations among each set of RNA-seq data generated. The results from the PCA, encompassing two biological replicates each for a total of eight data sets, showed that the data points associated with the Wild type clustered together, as did those associated with the Mutant type. Furthermore, the repetitions within each group also displayed strong correlations. This demonstrates that the generated data are reliable, as they show consistent patterns of correlation within the respective groups, thereby validating our experimental design and its outcomes (Figure 1). Among the produced sequencing datasets, only reads with a phred Quality Score above Q20 were used by filtering with BBduk. Filtered reads were aligned to *S. bicolor* BTx623 v3.0 from the NCBI using TopHat2. The processed reads for the untreated wild-type group had a total of 50,507,574 reads with 47,488,732 mapped reads, resulting in a mapping rate of 94%. Furthermore, the processed reads for the salt-treated wild-type group had a total of 41,265,572 reads with 38,694,601 mapped reads, resulting in a mapping rate of 93.77%. The processed reads for the untreated mutant group had a total of 42,181,414 reads with 40,151,012 mapped reads, resulting in a mapping rate of 95.18%. Finally, the processed reads for the salt-treated mutant group had a total of 55,522,938 reads with 51,585,251 mapped reads, resulting in a mapping rate of 92.91% (Table 2).

### 2.3. Comparative Analysis of Transcriptional Expression Patterns between the Two Sorghum Lines

**Figure 2:**
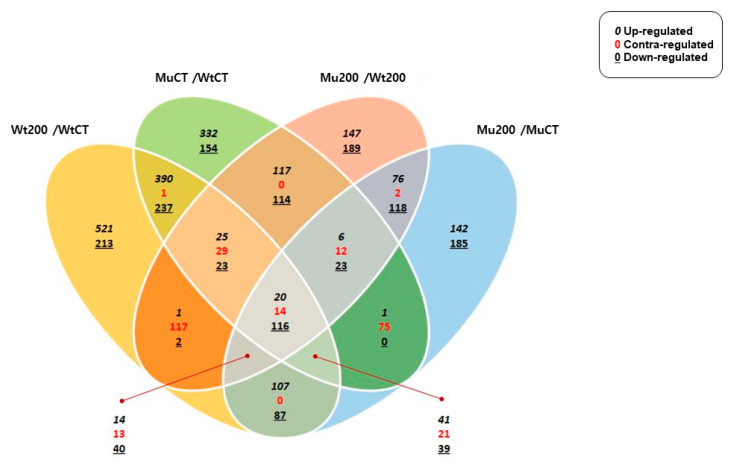
Differential gene expression analysis was conducted by venn diagram. WtCT, Wild type control; Wt200, Wild type 200 mM NaCl treatment; MuCT, Mutation Control; Mu200, Mutation 200 mM NaCl treatment. Contra-regulated genes refers to genes that exhibit changes in expression in opposite directions under different conditions or experimental treatments.

We obtained the transcriptomic information of the sorghum wild type and mutant under salt stress from the leaves. Differential gene expression analysis was conducted by comparing three conditions: wild type 200 mM NaCl treatment/control (Wt200/WtCT), mutant 200 mM NaCl treatment/control (Mu200/MuCT), and mutant 200 mM NaCl treatment/wild type 200 mM NaCl treatment (Mu200/Wt200). The selection of DEGs was conducted based on the following criteria: a fold change of 2.00, normalized data (log2) 4.00, and a *p*-value of 0.05. As a result, 582 DEGs were identified in Mu200/MuCT, including 263 up-regulated and 319 down-regulated DEGs. In WT200/WtCT, 1062 DEGs were identified, including 603 up-regulated, and 459 down-regulated DEGs. In Mu200/WT200, 510 DEGs were identified, including 219 up-regulated and 291 down-regulated DEGs (Figure 2). These DEGs are potential candidate genes that confer salt tolerance. We hypothesized that the DEGs in Mu200/Wt200 are associated with salt tolerance, and we analyzed these DEGs to investigate their association with salt tolerance.

### 2.4. Information on Variants in S. bicolor

We used RNAseq data of the wild type and mutant lines to identify variants that confer salt tolerance to sorghum. Variant calling was performed using sorghum BTx623 as a reference, and variants were classified according to their function, location, and effect by SnpEff. A total of 121,308 variants were identified in the mutant, occurring at a frequency of one per 5668 bases. This included 113,799 SNPs, 3142 insertions, and 4367 deletions. In contrast, a total of 99,911 variants were identified in the wild type with a frequency of one variant per 6883 bases.

We categorized the variants into four groups based on their overall impact: high, moderate, modifier, and low. High-impact variants directly affect gene function through a stop codon gain or loss, which likely results in significant changes in protein functionality due to transcript point mutations. Low-impact variants mostly include synonymous SNPs (sSNPs) that change the base sequence but not the amino acids, having minimal impact on the protein expression. Moderate-impact variants, known as missense variants, affect protein function through changes in the amino acids. Variants with a modifier impact affect gene function and include untranslated regions (3′ and 5′ UTRs) and intergenic variants [14]. In the mutant, the impact of the variants was as follows: modifier 87.591%, low 5.667%, moderate 6.192%, and high 0.549%. Additionally, in the wild type, the impact of the variants was classified as follows: 88.256% modifiers, 6.068% low impact, 5.331% moderate impact, and 0.344% high impact (Table 3).

We also classified the functional class of the variants according to their effects. The mutant had 47,530 nsSNPs and 38,839 sSNPs with an nsSNPs/sSNPs ratio of 1.2238. In contrast, the wild type had 33,502 nsSNPs and 34,125 sSNPs with an nsSNPs/sSNPs ratio of 0.9817 (Table 4).

### 2.5. Identification of Significantly Enriched Terms through Gene Onotology (GO) Analysis of the DEGs

We conducted a differential gene expression analysis on *S. bicolor* under a 200 mM salt treatment comparing the mutant and the wild type plants. A total of 510 DEGs were identified, including 219 upregulated and 291 downregulated DEGs. To investigate the impact of the nsSNPs on the expression of the DEGs, we compared the mutant and wild type plants with reference genomes, respectively, and identified the nsSNPs for each DEG. We identified 53 DEGs with nsSNPs among the 219 up-regulated DEGs (Table 5) and 93 DEGs with nsSNPs among the 291 down-regulated DEGs (Table 6). The difference in the expression of the DEGs between the two lines can vary depending on the occurrence of the nsSNPs.

We performed GO analysis on the DEGs with the nsSNPs using PlantRegMap. Based on the non-redundant GO annotation sets generated for 165 species, we conducted Fisher’s exact tests to identify significantly enriched GO terms for the input gene set. A built-in ID mapping tool was used to map the input IDs to the genome annotation IDs. The GO analysis of the up-regulated genes revealed that nine ‘Biological Processes’ were significantly enriched. These GO terms included the following: GO:0010132, dhurrin biosynthetic process; GO:0019756, cyanogenic glycoside biosynthetic process; GO:0042341, cyanogenic glycoside metabolic process; GO:0050898, nitrile metabolic process; GO:0080028, nitrile biosynthetic process; GO:1901806, beta-glucoside biosynthetic process; GO:1901804, beta-glucoside metabolic process; GO:0016137, glycoside metabolic process, and GO:0016138, glycoside biosynthetic process. On the other hand, the GO analysis of the downregulated genes revealed only one significantly enriched term in the ‘Molecular Function’ category, which is GO:0003824, catalytic activity (Table 7).

### 2.6. Confirmation of the Molecular Interaction Network of the Candidate Genes with Kyoto Encyclopedia of Genes and Genomes (KEGG) Pathway Analysis

**Figure 3:**
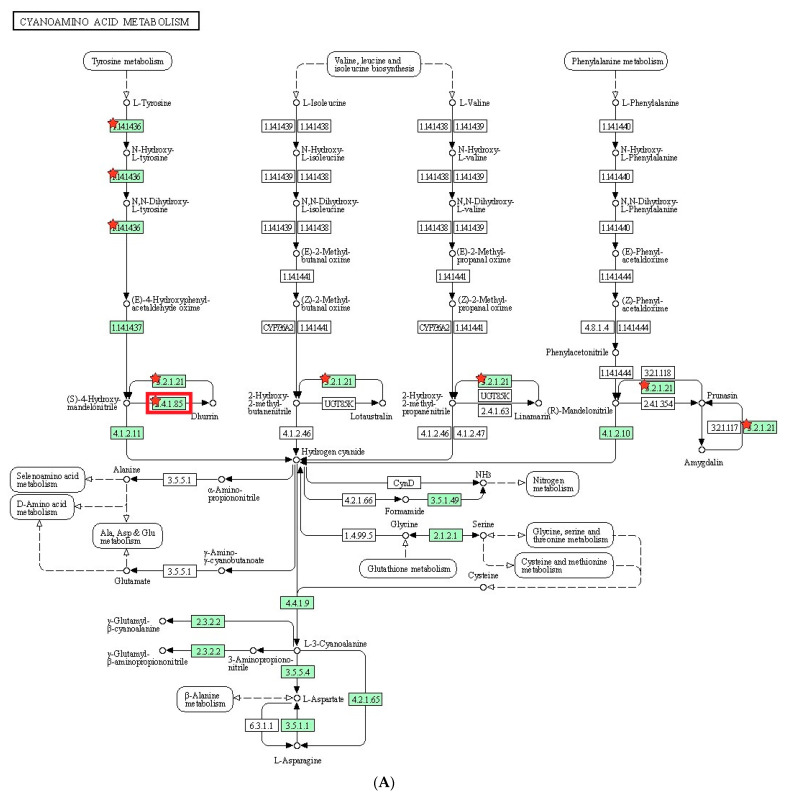

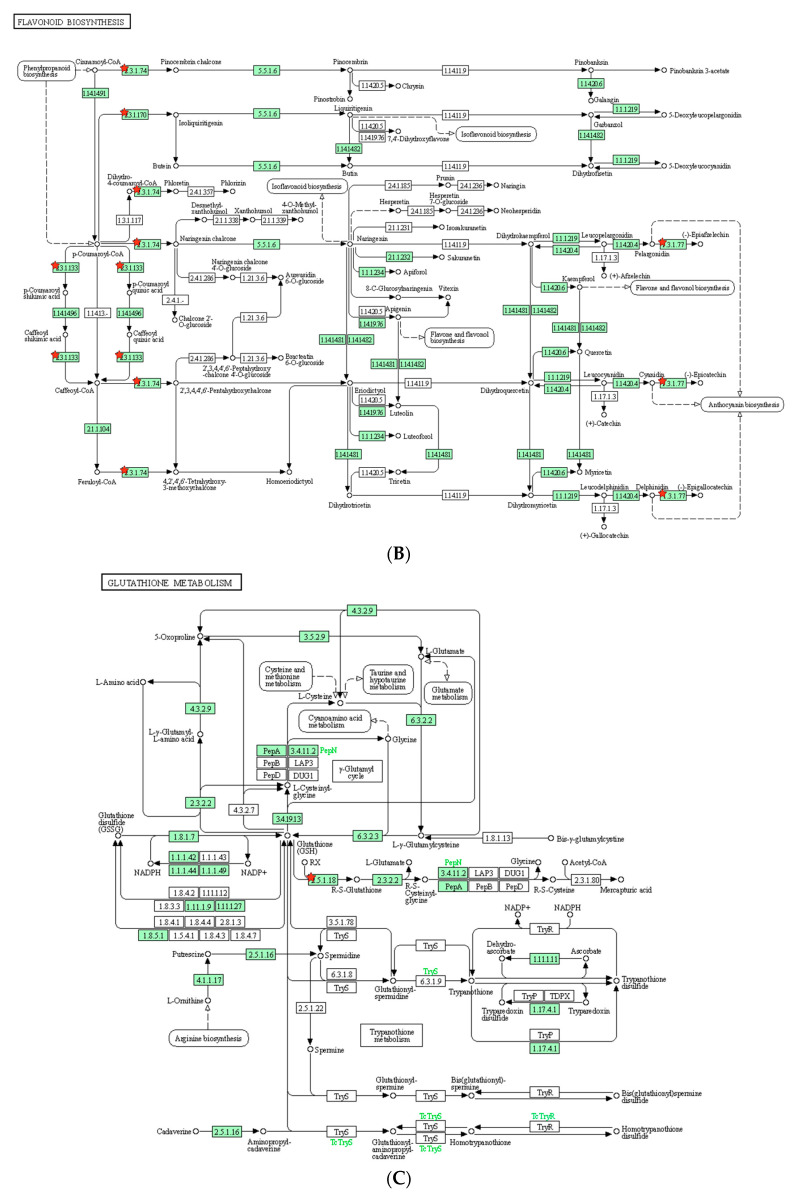
The diagram of the KEGG pathways (**A**) Cyanoamino acid metabolism; (**B**) Flavonoid biosynthesis; (**C**) Glutathione biosynthesis. The red rectangle indicates the pathway containing *LOC8060874* (cyanohydrin beta-glucosyltransferase).

We performed KEGG pathway analysis to identify molecular pathways potentially involved in the biological process of interest. Similar to the GO analysis, we performed KEGG pathway analysis on the DEGs between the mutant and wild type plants under salt stress treatment (Table 8). Several significantly enriched pathways were identified with metabolic pathway being the most common in both the up-regulated and down-regulated DEGs, including 15 and 17 genes, respectively. Other significantly enriched pathways included biosynthesis of secondary metabolites, which had 13 genes in the up-regulated DEGs and 12 genes in the down-regulated DEGs. In addition, cyanoamino acid metabolism was identified in the up-regulated DEGs, while flavonoid biosynthesis and glutathione metabolism were identified in the down-regulated DEGs (Figure 3). These findings provide valuable insights into the molecular mechanisms underlying the response to salt stress and highlight potential targets for further investigation. Several candidate genes of interest were found to be involved in these pathways, suggesting their potential functional relevance in the biological process under investigation. The molecular interaction/reaction network of the candidate genes in these pathways was also examined to further elucidate their potential roles.

## 3. Discussion

Salinity stress is one of the major abiotic stress factors that limit crop productivity worldwide. High salt concentrations in the soil can lead to ionic and osmotic imbalances in plants, resulting in reduced water uptake, nutrient deficiency, and cell damage. These physiological effects ultimately impair plant growth and development, resulting in decreased crop yields and quality. To address these challenges, developing salt-tolerant crops has become increasingly important. By understanding the mechanisms of the salt stress response in plants, researchers can identify genes and molecular pathways that contribute to salinity tolerance [1]. This knowledge can be used to develop breeding strategies that produce crops with improved salinity tolerance, thus increasing agricultural productivity in saline soils and helping to ensure global food security. Sorghum is an important crop in Korea because it can grow well in marginal lands, such as reclaimed tidal lands. However, these lands are often saline, which can limit crop growth and productivity. Therefore, developing sorghum cultivars with salt tolerance is essential for sustainable agriculture in these areas. By breeding sorghum with an enhanced salt tolerance, we can ensure stable crop yields and food security, even in challenging environments such as marginal lands. Moreover, sorghum’s small genome size of approximately 730 Mb makes it an appealing model for functional genomics of Saccharinae and other C4 grasses [10]. Despite its tolerance to salt stress, only a limited number of salt-responsive genes have been identified in sorghum.

In this study, we used the transcriptome of sorghum to examine differential gene expression. The mutation used in this study was generated by gamma-ray irradiation of seeds and selected as a salt-tolerant individual through field tests (the selection data are not provided in this paper). We hypothesized that the DEGs between the mutant and wild type under salt stress conditions may correspond to salt tolerance-related genes. To gain a more comprehensive understanding of the transcriptome expression, we also examined the impact of nsSNPs. The nsSNPs are genetic variations that result in a change in the amino acid sequence of a protein encoded by a gene. These variations can affect the structure and function of the protein, and therefore may have downstream effects on biological processes such as gene expression. In the context of DEGs, nsSNPs may be associated with differential gene expression between different samples or conditions. For example, if nsSNPs result in a protein variant that interacts differently with other proteins or regulatory factors, it could lead to changes in the gene expression levels or patterns. This, in turn, could impact cellular processes and phenotypic traits. Furthermore, nsSNPs can be used as markers for studying genetic variations and diversity in populations. By analyzing the distribution of nsSNPs in DEGs across populations, researchers can gain insights into the evolutionary history of different populations and how genetic variations may contribute to adaptation and disease susceptibility [36].

**Figure 4:**
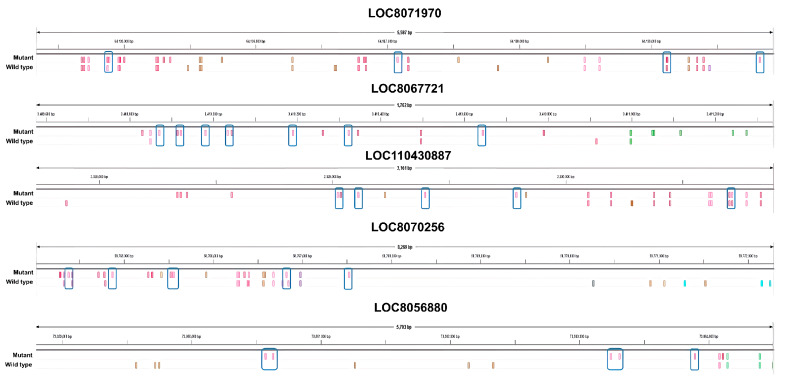
Identifying the location of nsSNPs that only occurred in the mutants compared to the wild type in five candidate genes. The blue rectangle indicates the location where nsSNPs occurred.

Specifically, we identified genes with a large difference in the number of nsSNPs between the mutant and wild type plants. We selected several candidate genes that showed a large difference in the nsSNPs (Figure 4). Based on our analysis, the *LOC8071970* gene, which encodes for the subtilisin-like protease SBT3.9 and is up-regulated in response to salt treatment, has the highest number of nsSNPs among all the up-regulated genes. Specifically, the mutant plant was found to have nine nsSNPs in *LOC8071970*, while the wild type plant had five nsSNPs. These differences in nsSNPs may be responsible for the up-regulation of the gene in the mutant in response to salt treatment. Subtilisin-like proteases, also known as subtilases, are a diverse family of serine peptidases found in many organisms, primarily in plants. They have a wide range of biological roles, including protein turnover, plant development, and interactions with the environment. Subtilases are known for their involvement in plant responses to both abiotic and biotic environmental stimuli [37,38,39]. Previous studies reported that subtilases are involved in drought and salt tolerance mechanisms following abiotic stimuli. For example, the *Arabidopsis thaliana* subtilase AtSBT6.1 was well-characterized for its association with the unfolded protein response during salt stress through the cleavage of an ER-resident type II membrane protein (bZIP28) [40].

*LOC8067721* functions as an acyl transferase 15. In the mutations, eight nsSNPs were expressed, which is eight times higher than the wild type. Numerous proteins, potentially acylated, have been pinpointed as contributors to processes such as vesicle trafficking, signal conveyance, primary and secondary metabolic functions, as well as responses to stress [41,42]. Overexpression of the Glycerol-3-phosphate acyltransferase (GPAT) gene in Arabidopsis plants resulted in increased salt tolerance at the germination stage with higher germination rates and longer root lengths than those of the wild type plants under salt stress conditions. Additionally, in the seedling stage, overexpressed plants showed a decrease in the chlorophyll content, and the unsaturated fatty acid content of phosphatidylglycerol (PG) decreased less than in the wild type plants under salt stress. These results suggest that GPAT plays a role in the production of unsaturated fatty acids in PG, which are important for maintaining membrane fluidity and function under salt stress. Therefore, the overexpression of GPAT could enhance plant salt tolerance by increasing the production of unsaturated fatty acids and improving membrane function under high salinity conditions [43].

*LOC110430887* is a down-regulated DEG that functions as cysteine-rich receptor-like (CRK) protein kinase 10. In the mutants, eight nsSNPs were detected, which is five more than the three nsSNPs in the wild type. CRKs undergo transcriptional induction under abiotic stress conditions, including exposure to salicylic acid, ozone, UV light, as well as during drought and salt treatments [44,45,46]. Another study reported that a specific type of receptor-like kinase, called CRK5, is involved in abscisic acid (ABA) signaling in *Arabidopsis thaliana*. Overexpression of CRK5 increased ABA sensitivity and enhanced plant drought tolerance without affecting plant growth or productivity. The loss-of-function mutation of the CRK5 gene did not affect the ABA response, while overexpression of two homologs of CRK5 conferred ABA responses, suggesting redundancy [47].

*LOC8070256* is a protein that functions as a linoleate 9S-lipoxygenase. In the mutant, 12 nsSNPs were detected, which is five more than the seven found in the wild type. Lipoxygenases (LOXs) are enzymes that occur naturally and are found widely in plants and animals. LOX enzymes, which are essential for plant growth and development, are the products of a multigene family. There is a noted correlation between enhanced LOX activity and improved salt tolerance [48,49]. These enzymes can be non-sulfur iron, non-heme iron, or manganese-containing dioxygenase redox enzymes. LOXs catalyze the oxidation of polyunsaturated fatty acids into fatty acid hydroperoxides. This oxidation process converts linolenic acid, a precursor in the biosynthesis of jasmonic acid (JA), into 12-oxo-phytodienoic acid, which is carried out through oxygenation with LOX, allene oxide synthase, and allene oxide cyclase. JA is involved in various physiological and biochemical processes such as seed germination, fruit ripening, senescence, and others. Additionally, LOXs play a vital role in defense responses against biotic stress, including pests, insects, and pathogenic attacks and abiotic stress such as wounding, extreme temperatures, UV-rays, oxidative stress, and drought [50].

*LOC8056880* is a functional sugar transport protein 13. Six nsSNPs were detected in the mutant, which is six times higher than in the wild type. SWEETs (Sugars Will Eventually be Exported Transporters) are a family of sugar transporters that facilitate the diffusion of sugar molecules across cell membranes. In plants, SWEETs play important roles in various physiological processes including phloem loading, senescence, pollen nutrition, grain filling, nectar secretion, and abiotic and biotic stress regulation. SWEET transporters are involved in plant development and abiotic stress as well as in their gene expression dynamics under different stresses in different plant species. SWEET could lead to improvements in crop productivity and stress tolerance [51].

To understand the metabolic processes in plants, we conducted KEGG pathway analysis on the DEGs. In the identified pathway, cyanoamino acid metabolism, flavonoid biosynthesis, and glutathione metabolism were reported to play a role in salt tolerance in plants. Cyanoamino acid metabolism is involved in the biosynthesis of cyanogenic glycosides, which are known to act as a defense mechanism against herbivores and pathogens. In addition, cyanogenic glycosides have been shown to help plants cope with salt stress by regulating ion homeostasis [52]. Flavonoid biosynthesis is a metabolic pathway that produces flavonoids, which are known to have antioxidant properties. Flavonoids have been found to be involved in salt stress tolerance by protecting plants against oxidative damage caused by high salt concentrations [53]. Glutathione metabolism is a crucial pathway for maintaining cellular redox homeostasis. Glutathione plays an important role in protecting cells from oxidative damage caused by salt stress. In addition, glutathione metabolism has been found to regulate ion transport and osmotic balance in response to salt stress [54]. Overall, these metabolic pathways are involved in regulating ion homeostasis, protecting against oxidative damage, and maintaining cellular redox balance, which are all important factors in salt stress tolerance in plants.

Based on the DEGs with nsSNPs, we conducted GO analysis to identify significant GO terms. Out of the nine significantly enriched GO terms for up-regulated genes, it was identified that seven GO terms were related to glycosides. Interestingly, three of these terms were specifically connected to cyanogenic glycosides, including the dhurrin biosynthetic process (GO:0010132), cyanogenic glycoside biosynthetic process (GO:0019756), and cyanogenic glycoside metabolic process (GO:0042341). Cyanogenic glycosides are a class of secondary metabolites found in various plant species. They contain a cyanide group bonded to a sugar molecule and can release toxic hydrogen cyanide when plant tissue is damaged. Dhurrin is one of the most well-studied cyanogenic glycosides and is predominantly found in *S. bicolor* and other related plant species [55]. Osmotic stress is a form of environmental stress that plants experience when there is an imbalance in water potential between the plant cells and their surroundings. This can be due to high salt concentrations, drought conditions, or even low temperatures. Osmotic stress can lead to cellular dehydration and negatively impact plant growth and development [56]. The relationship between cyanogenic glycosides such as dhurrin and osmotic stress is complex and multifaceted. During osmotic stress, plants are more vulnerable to damage; therefore, accumulating cyanogenic glycosides may serve as an additional layer of protection. Some research suggests that cyanogenic glycosides might also function as osmoprotectants, helping plants to tolerate osmotic stress. They might do this by stabilizing proteins and cellular structures, reducing cellular damage caused by stress, and maintaining cellular water balance. Plant exposure to osmotic stress may lead to changes in the accumulation patterns of cyanogenic glycosides including dhurrin. These changes may result from stress-induced alterations in the expression of genes involved in the biosynthesis and metabolism of cyanogenic glycosides. This can lead to a differential distribution of these compounds within the plant, potentially as a response to stress conditions [57,58,59]. Although the relationship between cyanogenic glycosides such as dhurrin and osmotic stress is not yet fully understood, it appears that they might play a role in plant defense mechanisms, possibly act as osmoprotectants, and have altered accumulation patterns under stress conditions.

**Figure 5:**
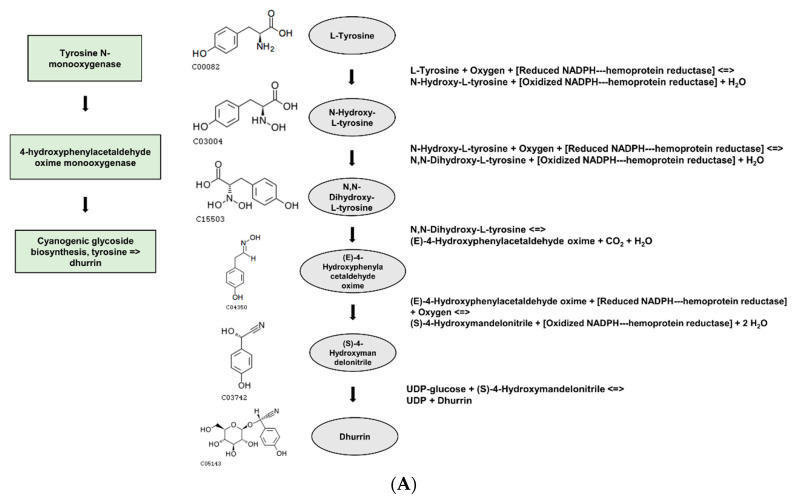

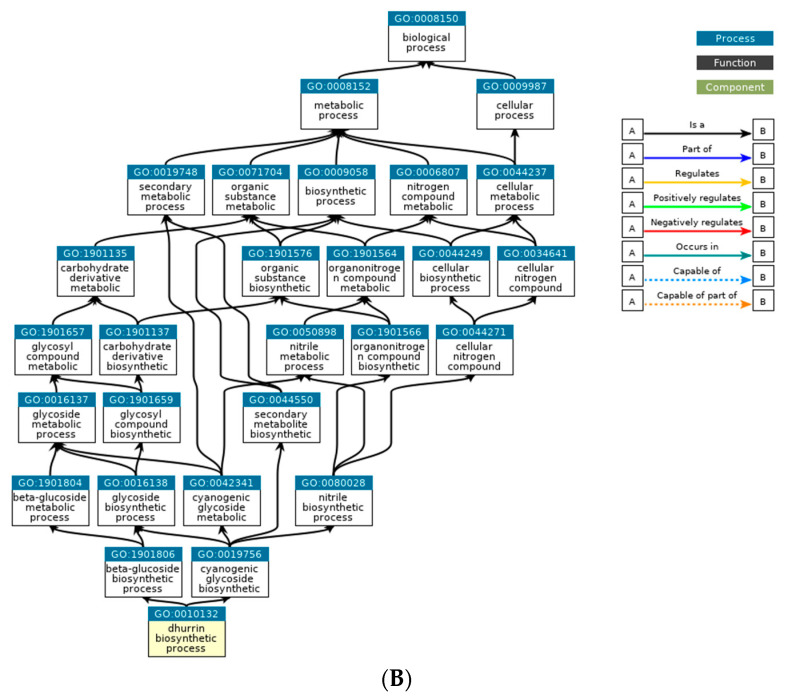
(**A**), Module of *LOC8060874* (cyanohydrin beta-glucosyltransferase), sbi_M00369 Cyanogenic glycoside biosynthesis, tyrosine => dhurrin (**B**), Ancestor chart for GO:0010132, the dhurrin biosynthetic process.

The gene *LOC8060874* (cyanohydrin beta-glucosyltransferase), which falls under the GO term: dhurrin biosynthetic process (GO:0010132), had a single nsSNP and higher gene expression levels in the gamma-ray-induced salt-tolerant mutant compared to the wild type under salt stress conditions (Table 5 and Figure 5). The nsSNP in *LOC8060874* leads to an amino acid substitution from alanine to aspartic acid, altering the protein’s chemical properties due to the differences between the non-polar, hydrophobic alanine and the polar, negatively charged aspartic acid due to its carboxyl side chain (Figure 5). This difference in chemical properties could potentially affect the protein’s folding, stability, or interaction with other molecules, which might be one of the contributing factors for the observed salt tolerance in the mutant plant, considering the role of dhurrin under osmotic stress conditions [10,60].

Several mechanisms were proposed to help plants lower the concentrations of these detrimental free radicals. One such mechanism involves increasing the production of secondary metabolites such as phenolics and cyanogenic glucosides [61]. This increase could be a result of diverting excess NADPH^+^, which accumulates due to stomatal closure and reduced CO_2_ flux. There may also be a more direct association. For instance, previous research reported that the production of amides derived from cyanogenic glucosides rises with increasing H_2_O_2_ levels [62]. The proposed dhurrin turnover pathway in sorghum also implies amide formation, suggesting a possible role for dhurrin in mitigating ROS-mediated stress. Therefore, amide formation from cyanogenic glucosides might serve as a non-enzymatic scavenger mechanism in cyanogenic plants, helping them cope with oxidative stress and improve their drought tolerance [63].

Moreover, recent research suggests that dhurrin may play a role in plant growth and development in addition to defense. Sorghum generates the cyanogenic glucoside known as dhurrin. In the early stages of growth, young sorghum plants contain high concentrations of dhurrin, which gradually decrease as the plant matures [64]. Three-day-old seedlings of sorghum can have dhurrin concentrations as high as 6% with the highest concentration localized in the growing shoot tips where it may account for up to 30% of the total shoot dry weight [65]. Moreover, a previous study reported that plants lacking dhurrin tend to be smaller compared to their wild-type counterparts. The absence of dhurrin appears to hinder growth during early developmental stages. Furthermore, plants deficient in dhurrin take longer to mature, potentially due to delayed flowering. Dhurrin generation may benefit plant growth, particularly during critical growth stages such as germination, by providing a readily available source of reduced nitrogen [66]. This is consistent with our research findings, which show that the germination rate of the mutant plants was significantly higher than that of the wild-type plants. The increase in dhurrin expression and subsequent elevation in dhurrin levels might be closely related to the initial germination and growth stages (Table 1).

**Figure 6:**
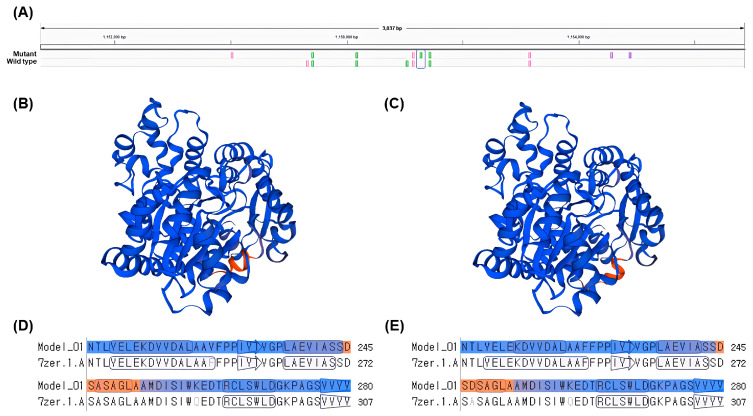
Prediction of the 3D protein sequence structure for dhurrin biosynthesis-associated gene *LOC8060874*. (**A**) comparison of nsSNP between wild type and mutant, (**B**) prediction of protein structure for wild type, (**C**) prediction of protein structure for mutant, (**D**) amino acid sequence of wild type, (**E**) amino acid sequence of mutant.

However, it is important to note that this study does not provide definitive evidence that the gene *LOC8060874* (cyanohydrin beta-glucosyltransferase) directly confers salt tolerance. Instead, it suggests that the gene could be linked to the salt tolerance phenotype through its involvement in the dhurrin biosynthesis process. While there have been several studies exploring the relationship between dhurrin and osmotic stress in plants, it has been difficult to establish a clear correlation. This research proposes a possible connection between dhurrin and osmotic stress, particularly salt tolerance, in plants from the perspective of amino acid changes (Figure 6). This study highlights the potential significance of amino acid substitutions in determining the function of proteins involved in dhurrin biosynthesis and suggests that leveraging such genetic variations could improve crop performance under stress conditions, such as high salinity. Further investigation into the molecular mechanisms underlying this salt tolerance and the specific structural changes in the protein could provide valuable insights into plant stress response pathways and help in the development of more resilient crop varieties. Additionally, generating transgenic plants expressing the mutated gene and assessing their salt tolerance would provide more conclusive evidence for the role of this amino acid change in contributing to salt tolerance.

Our results suggest that the differences in nsSNPs between the mutant and wild type plants may have a significant impact on the expression of DEGs and may contribute to salt tolerance in *S. bicolor*. The differential expression of genes in response to the difference in nsSNPs between the mutant and wild type plants suggests that these nsSNPs may have an impact on the salt tolerance of *S. bicolor*. Specifically, we observed that several genes showed a significant change in expression level with some genes being up-regulated and others being down-regulated. These findings suggest that nsSNPs may play a crucial role in the regulation of gene expression and the plant’s ability to withstand salt stress. In light of our findings, we suggest that further in-depth study of the candidate genes is critical. Subsequent studies, especially those employing reverse genetics experiments, could provide definitive insights into these genes’ roles in the salinity tolerance of *S. bicolor*. The impacts of nsSNPs on gene expression and salt tolerance could be more comprehensively understood through such rigorous validation. This research could guide the breeding of resilient *S. bicolor* cultivars for saline environments. Consequently, this study could potentially provide a foundation for the development of new varieties to prepare for future food crises.

## 4. Materials and Methods

### 4.1. Plant Materials & Experimental Design

In this study, the *S. bicolor* accession (IT124115, wild type) and a gamma ray mutant line (IT124115M) were used as the plant materials. The mutant line was created by exposing the seeds to 250 Gy of gamma radiation, and the surviving individuals in an environment with a salinity of 2% (about 0.342 M NaCl concentration) were considered to have salt tolerance [34]. The seeds harvested from these surviving individuals were used for the experiment. Prior to the test, all seeds were sterilized with a thiram-benomyl mixture (5 g/L) for 24 h. Plant growth and stress treatments were conducted at Chungnam National University, South Korea (36°22′06.6′′ N 127°21′11.3′′ E) from May to June 2022. The seeds were germinated at a temperature of 26 °C in a growth chamber and then grown at the same temperature with a light/dark cycle of 16/8 h. Seeds of both varieties were initially grown in plastic pots (6 cm deep with a 1.5 cm diameter) filled with bed soil and then transferred to new pots (8 cm deep with a 7 cm diameter) filled with sterilized vermiculite until they reached the seedling stage (with four-five leaves and a height of 15–20 cm). Half-strength Hoagland nutrient solution was added to a reservoir, and the plant roots absorbed the solution through the vermiculite without directly touching the solution. The plants were grown for seven days in a normal half-strength Hoagland nutrient solution before the stress treatment. Uniformly sized plants were selected for the experiment. The stress treatment was performed with a half-strength Hoagland nutrient solution containing 200 mM sodium chloride with a non-treated control. The leaves of the plants were sampled 48 h after the stress treatment with three biological replicates.

### 4.2. Phenotyping and Data Analysis

To investigate the phenotypic information of the wild type and mutant *S. bicolor* prior to the genomic analysis, we measured the germination rate, leaf length, and leaf number. For the germination rate, we sowed 50 seeds of each type of sorghum and counted the number of germinated seedlings just before the stress treatment expressed as a percentage. Additionally, to measure the leaf length and number, we selected 15 plants showing an average growth and measured the length of the longest leaf from the top of each plant and counted all the leaves that had grown.

### 4.3. Statistical Analysis

The phenotypic data were presented as the mean value obtained from 15 independent biological replicates, accompanied by the corresponding standard error of the mean (SEM). For the purpose of statistical analysis, a One-way ANOVA was performed using IBM SPSS Statistics version 24 software (IBM SPSS, Inc., Armonk, NY, USA).

### 4.4. RNA Extraction and mRNA-Sequencing

RNA extraction was performed on both wild type and mutant *S. bicolor* leaves using Trizol reagent (Invitrogen, CA, USA) followed by quality assessment with the TapeStation4000 System (Agilent Technologies, Amstelveen, The Netherlands) and quantification with the ND-2000 Spectrophotometer (Thermo Inc., Waltham, MA, USA). RNA samples with a concentration of at least 100 ng/μL and a 260/280 ratio within 1.8–2.0 were selected for sequencing. To construct libraries, the NEBNext Ultra II Directional RNASeq Kit (NEW ENGLAND BioLabs, Inc., Ipswic, MA, USA) was used with the Poly(A) RNA Selection Kit (LEXOGEN, Inc., Austria). The manufacturer’s instructions were followed for cDNA synthesis and shearing, and Illumina indexes 1–12 were used for indexing prior to the PCR enrichment. Library quantification was performed using the StepOne Real-Time PCR System (Thermo Inc., Waltham, MA, USA), and the mean fragment size was determined using the TapeStation HS D1000 Screen Tape (Agilent Technologies, Amstelveen, The Netherlands). High-throughput paired-end 100 sequencing was carried out using the NovaSeq 6000 (Illumina, Inc., San Diego, CA, USA) with two biological replicates for each treatment and accession. The cDNA synthesis and shearing steps were carried out following the manufacturer’s instructions, and Illumina indexes 1–12 were used for indexing before PCR enrichment. Library quantification was performed with the StepOne Real-Time PCR System (Thermo Inc., Waltham, MA, USA), while the mean fragment size was determined using the TapeStation HS D1000 Screen Tape (Agilent Technologies, Amstelveen, The Netherlands). High-throughput sequencing was performed using the NovaSeq 6000 (Illumina, Inc., San Diego, CA, USA) with paired-end 100 sequencing.

### 4.5. mRNA-Sequencing Data Analysis

Quality control was performed on the raw sequencing data with FastQC. Reads with adapter contamination and low-quality scores (<Q20) were removed with FASTX_Trimmer and BBMap. The processed reads were then mapped to the reference genome of *S. bicolor* BTx623 v3.0 from the NCBI with TopHat2 [67]. The read count data was normalized using the FPKM+Geometric normalization method with EdgeR in R. The FPKM values were estimated with Cufflinks. Finally, data mining and graphical visualization were performed with ExDEGA. DEGs were filtered based on the fold change (FC) being equal to or greater than two, the normalized data (log_2_) being equal to or greater than four, and a *p*-value of 0.05 or less. The annotation of confirmed DEGs was performed using BlastX against the NCBI Plant Nr Database with a cut-off E-value of e^−5^.

### 4.6. GO Analysis

GO analysis on DEGs was performed between the wild type and mutant *S. bicolor* plants under salt stress conditions with PlantRegMap “http://plantregmap.gao-lab.org/ (accessed on 21 March 2023)”. The enriched GO terms were identified using single enrichment analysis with the background gene set as the whole genome of *S. bicolor* and the query gene set as the DEGs under salt stress conditions between the wild type and mutant plants. The enriched GO terms were classified into three categories: biological process, cellular component, and molecular function [68]. The topGO (v2.22) was used with the sets of non-redundancy GO annotations for 165 species, and Fisher’s exact tests were performed to identify significantly enriched GO terms for the input gene set. The FDR values used a *p*-value threshold of 0.05 to determine significant GO terms.

### 4.7. KEGG Pathway Analysis

KEGG pathway analysis was performed to identify the biological pathways associated with DEGs under salt treatment between the *S. bicolor* wild type and mutant. The analysis involved mapping a set of genes to the KEGG pathway database, which contains information about metabolic and signaling pathways for various organisms. The KEGG web server, available at “http://www.genome.jp/kegg/ (accesed on 11 March 2023)”, was used to assign the KEGG pathways to the genes.

### 4.8. Variant Calling

To call SNPs for the wild type and mutant *S. bicolor*, each RNAseq data was mapped to the BTx623 sorghum reference genome using TopHat2 [67]. Bcftools was used to identify SNPs from the BAM files and generate variant calling files (VCFs) containing the SNPs. Then, SnpEff v4.3 was used to annotate the SNPs with information such as gene names, functional effects, and population frequencies. An annotated VCF file generated by SnpEff was used for visualizing the positions of the SNPs using Integrative Genomics Viewer.

### 4.9. Protein Modeling

We created a new reference sequence incorporating variant information using the generated VCF file and the bcftools consensus tool. Based on this new reference, we extracted the target gene sequences using bedtools. We then used the extracted nucleotide sequences to identify the amino acid sequences with the NCBI ORF Finder. Upon verifying the amino acid sequences, we utilized the Swiss-Model to examine the three-dimensional structure of the protein based on the amino acid sequence [69].

## 5. Conclusions

This study focused on identifying genes and nsSNPs associated with salt tolerance in *S. bicolor*, by comparing the transcriptomes of a wild type and a mutant line under salt stress conditions. The mutant lines, created through gamma radiation and having survived a salinity field test, exhibited significantly different gene expression patterns compared to the wild type. DEGs were involved in various metabolic processes such as cyanoamino acid metabolism, flavonoid biosynthesis, and glutathione metabolism, all critical to salt stress tolerance in plants. In order to validate these genes definitively, additional reverse genetics studies will be necessary. Several candidate genes were selected based on a large difference in nsSNPs and altered gene expression in the mutant lines. These genes play roles in plant development, stress regulation, and environmental interactions. Furthermore, we noted a potential link between salt tolerance and a specific amino acid change from alanine to aspartic acid in dhurrin biosynthesis related genes. These findings provide a comprehensive understanding of the mechanisms underlying salt tolerance in sorghum and are a valuable resource for future sorghum genome studies and breeding programs aimed at improving salt tolerance. In conclusion, this study has significant implications for enhancing the sustainability and productivity of sorghum agriculture in regions affected by soil salinization.

## Figures and Tables

**Table 1:** Phenotype information of the two sorghum lines before the salt stress treatment.

	Germination Rate	Number of Leaves	Length of Leaves
Wild type	44%	4.8 ± 0.41	30.25 ± 3.55 cm
Mutant line	92%	4.93 ± 0.26	32.15 ± 3.85 cm

**Table 2:** Alignment information of the two sorghum lines.

	Processed Reads	Mapped Reads	Mapping Rate
WtCT	50,507,574	47,488,732	94%
Wt200	41,265,572	38,694,601	93.77%
MuCT	42,181,414	40,151,012	95.18%
Mu200	55,522,938	51,585,251	92.91%

WtCT, Wild type control; Wt200, Wild type 200 mM NaCl treatment; MuCT, Mutation Control; Mu200, Mutation 200 mM NaCl treatment.

**Table 3:** Number of variants by impact.

	Wild Type	Mutant
HIGH	2155 (0.334%)	4200 (0.549%)
LOW	37,974 (6.068%)	43,330 (5.667%)
MODERATE	33,363 (5.331%)	47,345 (6.192%)
MODIFIER	552,289 (88.256%)	669,691 (87.591%)

**Table 4:** The functional class of SNPs according to their effects in the two sorghum lines.

	Wild Type	Mutant
nsSNPs	33,502	47,530
sSNPs	34,125	38,839
nsSNPs/sSNPs ratio	0.9817	1.2238

**Table 5:** Up-regulated DEGs with nsSNPs in the two sorghum lines.

Gene	Description	Mu	Wt
*LOC8071970*	subtilisin-like protease SBT3.9	9	5
*LOC8067721*	acyl transferase 15	8	1
*LOC8079881*	CBS domain-containing protein CBSCBSPB3	7	5
*LOC8073431*	vacuolar amino acid transporter 1	6	1
*LOC8066963*	cytochrome P450 94C1	5	2
*LOC8068062*	UDP-glycosyltransferase 88B1	5	8
*LOC8083632*	protein NRT1/PTR FAMILY 8.3	5	9
*LOC110433972*	probable protein phosphatase 2C 1	4	2
*LOC110436757*	probable adenylate kinase 5, chloroplastic	4	2
*LOC8082973*	O-methyltransferase ZRP4	4	2
*LOC8058225*	probable isoprenylcysteine alpha-carbonyl methylesterase ICMEL2	4	3
*LOC8060874*	cyanohydrin beta-glucosyltransferase	4	4
*LOC110432978*	beta-amylase	4	5
*LOC8076352*	ABC transporter G family member 11	4	6
*LOC110435588*	beta-glucosidase-like SFR2, chloroplastic	4	11
*LOC8074133*	protein dehydration-induced 19 homolog 5	3	1
*LOC8070506*	beta-amylase 1, chloroplastic	3	1
*LOC8064623*	auxin-binding protein 4	3	1
*LOC8065646*	protein detoxification 40	3	2
*LOC8056874*	homocysteine S-methyltransferase 1	3	2
*LOC8065163*	cytochrome P450 89A2	3	2
*LOC8057564*	mitogen-activated protein kinase kinase kinase 2	3	3
*LOC8059087*	vacuolar cation/proton exchanger 1c	2	1
*LOC8055455*	UPF0235 protein C15orf40 homolog	2	1
*LOC8077931*	peptide chain release factor PrfB3, chloroplastic	2	1
*LOC8055150*	mitochondrial carnitine/acylcarnitine carrier-like protein	2	1
*LOC8065993*	inosine-5′-monophosphate dehydrogenase	2	1
*LOC8064978*	F-box/kelch-repeat protein SKIP25	2	1
*LOC8070845*	two-component response regulator-like PRR37	2	2
*LOC8073643*	tropinone reductase homolog At5g06060	2	2
*LOC8072215*	ervatamin-B	2	2
*LOC8073557*	agmatine deiminase	2	2
*LOC8076019*	RING-H2 finger protein ATL8	2	3
*LOC8056546*	peroxidase 25	2	5
*LOC8061413*	tyrosine N-monooxygenase	1	1
*LOC8069641*	transcription factor bHLH35	1	1
*LOC8084948*	probable protein phosphatase 2C 30	1	1
*LOC8057982*	probable calcium-binding protein CML48	1	1
*LOC8078462*	nitrile-specifier protein 5	1	1
*LOC8069505*	heavy metal-associated isoprenylated plant protein 33	1	1
*LOC8061953*	heat stress transcription factor C-2b	1	1
*LOC8065043*	dehydrogenase/reductase SDR family member 12	1	1
*LOC8069346*	chaperone protein dnaJ 8, chloroplastic	1	1
*LOC8078558*	CBL-interacting protein kinase 29	1	1
*LOC8067575*	asparagine synthetase	1	1
*LOC8064317*	serotonin N-acetyltransferase 2, chloroplastic	1	2
*LOC110430006*	non-symbiotic hemoglobin 1-like	1	2

Mu, number of nsSNPs in the Mutation; Wt, number of nsSNPs in the wild type.

**Table 6:** Down-regulated DEGs with nsSNPs in the two sorghum lines.

Gene	Description	Mu	Wt
*LOC8070256*	linoleate 9S-lipoxygenase	12	7
*LOC8084078*	cysteine-rich receptor-like protein kinase 6	9	6
*LOC8070443*	leaf rust 10 disease-resistant locus receptor-like protein kinase-like 2.7	9	8
*LOC110432221*	glucan endo-1,3-beta-glucosidase 3-like	9	9
*LOC110430887*	cysteine-rich receptor-like protein kinase 10	8	3
*LOC8056880*	sugar transport protein 13	6	1
*LOC8060773*	hydroxycinnamoyltransferase 4	6	1
*LOC8056023*	probable metal-nicotianamine transporter YSL12	6	4
*LOC8084623*	geraniol 8-hydroxylase	6	4
*LOC8069498*	1-aminocyclopropane-1-carboxylate oxidase	6	4
*LOC8083115*	indole-2-monooxygenase	6	7
*LOC8078145*	WRKY transcription factor 42	5	1
*LOC8065260*	AAA-ATPase ASD, mitochondrial	5	2
*LOC8057144*	3-oxo-Delta(4,5)-steroid 5-beta-reductase	5	2
*LOC8062818*	benzoate O-methyltransferase	5	4
*LOC8084079*	cysteine-rich receptor-like protein kinase 6	5	5
*LOC8076338*	putative disease resistance RPP13-like protein 3	5	11
*LOC8085100*	endo-1,3;1,4-beta-D-glucanase	4	2
*LOC8063962*	UDP-N-acetylglucosamine diphosphorylase 1	4	3
*LOC8060812*	lecithin-cholesterol acyltransferase-like 1	4	6
*LOC8068603*	primary amine oxidase 2	3	1
*LOC8069448*	nucleotide pyrophosphatase/phosphodiesterase	3	1
*LOC8068908*	MLO-like protein 15	3	1
*LOC8075402*	UDP-glycosyltransferase 85A2	3	2
*LOC8076184*	protein SRG1	3	2
*LOC110430289*	GDSL esterase/lipase At5g45910-like	3	2
*LOC110434959*	aldehyde dehydrogenase family 2 member B7, mitochondrial-like	3	2
*LOC8055178*	ABC transporter C family member 10	3	2
*LOC8069878*	pirin-like protein	3	3
*LOC8068813*	GDSL esterase/lipase At1g28650	3	3
*LOC8062072*	cytochrome P450 78A9	3	3
*LOC8070835*	cysteine-rich receptor-like protein kinase 25	3	3
*LOC8079442*	putative multidrug resistance protein	3	4
*LOC8070185*	magnesium/proton exchanger 1	3	4
*LOC8073263*	lysine histidine transporter 2	3	5
*LOC8076337*	wall-associated receptor kinase 2	3	14
*LOC8073798*	receptor-like protein kinase 5	3	17
*LOC8080791*	universal stress protein PHOS34	2	1
*LOC8059920*	serine carboxypeptidase 2	2	1
*LOC8055481*	putative serine/threonine-protein kinase-like protein CCR3	2	1
*LOC8063167*	probable glutathione S-transferase GSTU6	2	1

Mu, number of nsSNPs in the Mutation; Wt, number of nsSNPs in the wild type.

**Table 7:** DEGs with significantly enriched GO terms for the two sorghum lines.

GO.ID	Term	Count	Regulation
GO:0010132	dhurrin biosynthetic process	2	Up
GO:0019756	cyanogenic glycoside biosynthetic process	2	Up
GO:0042341	cyanogenic glycoside metabolic process	2	Up
GO:0050898	nitrile metabolic process	2	Up
GO:0080028	nitrile biosynthetic process	2	Up
GO:0016138	glycoside biosynthetic process	2	Up
GO:1901806	beta-glucoside biosynthetic process	2	Up
GO:1901804	beta-glucoside metabolic process	2	Up
GO:0016137	glycoside metabolic process	2	Up
GO:0003824	catalytic activity	50	Down

**Table 8:** KEGG pathways of the DEGs in the two sorghum lines.

Term	Count	Regulation
Biosynthesis of secondary metabolites	15/12	Up/Down
Metabolic pathway	13/17	Up/Down
Cyanoamino acid metabolism	3	Up
Flavonoid biosynthesis	3	Down
Glutathione biosynthesis	3	Down

## Data Availability

The data used in this study can be accessed at [https://www.ncbi.nlm.nih.gov/bioproject/952508 (accessed on 21 March 2023)].
